# Supplementary figures and images for: Repression of varicella zoster virus gene expression during quiescent infection in the absence of detectable histone deposition
Source: PLoS Pathog. 2025 Feb 10;21(2):e1012367. doi: 10.1371/journal.ppat.1012367 (PMC11838886; doi:10.1371/journal.ppat.1012367)

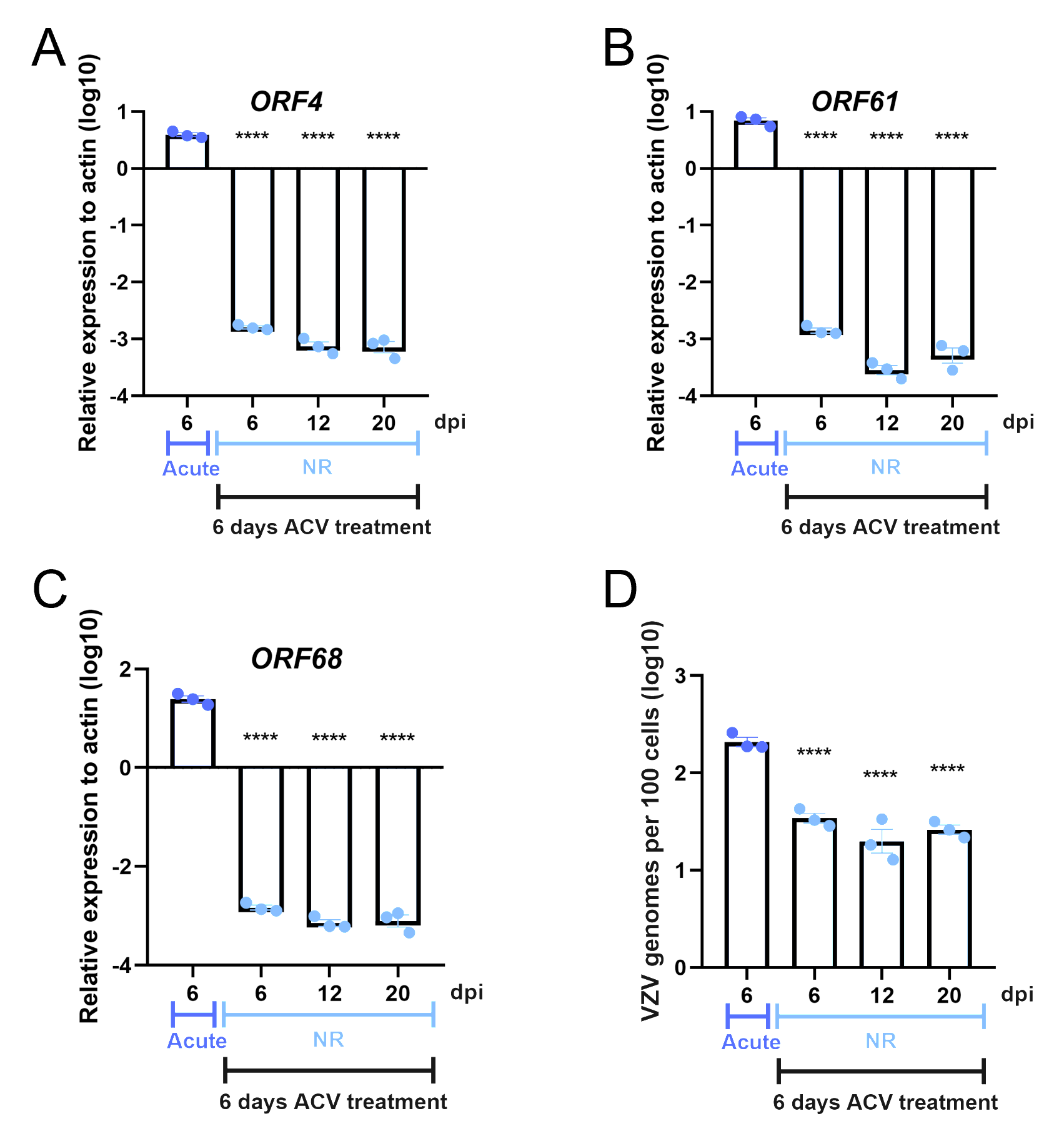

Supplement: S1 Fig — Relative gene expression of VZV genes, n=3 (A-C) and quantification of VZV genomes (D), n=3, in VZV Δ57-GFP infected dSH-SY5Y cells in the absence (acute) or presence of ACV for 6 dpi. Statistical comparisons between acutely infected cells and rest of the conditions were performed using one-way ANOVA on log-transformed raw data to stabilize variances and improve normality. P > 0.05 (ns), P ≤ 0.05 (*), P ≤ 0.01 (**), P ≤ 0.001 (***), P ≤ 0.0001 (****). Abbreviations: ACV, acyclovir (TIF) [file ppat.1012367.s001.tif]

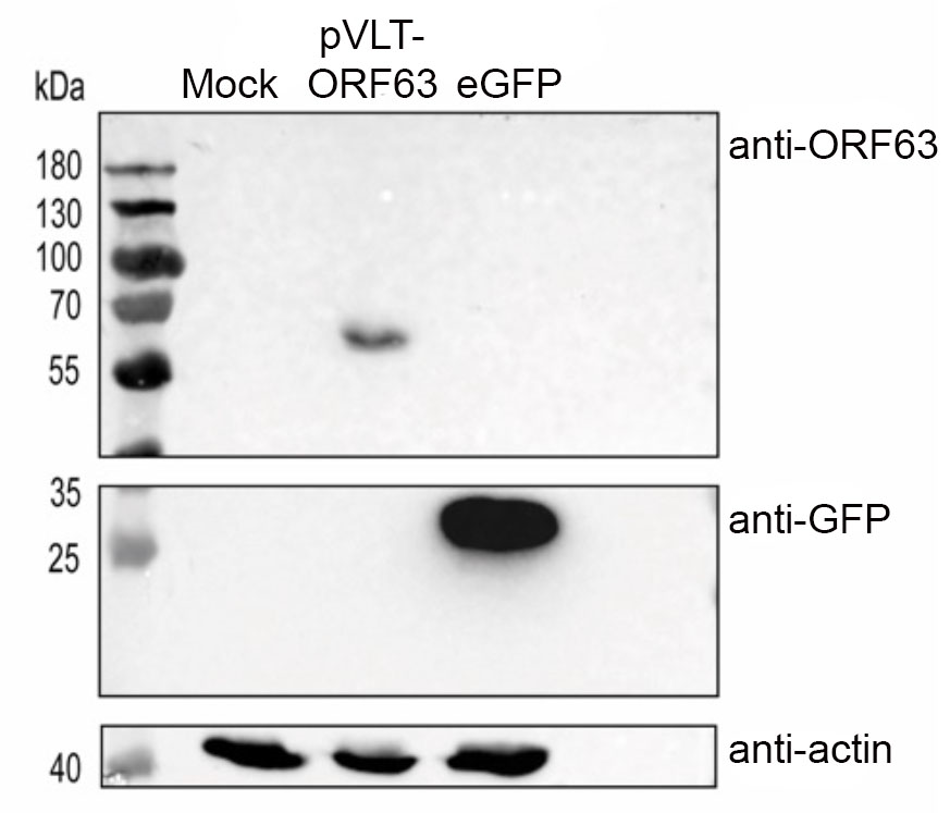

Supplement: S2 Fig — Western blot showing expression of pVLT-ORF63 (top) and eGFP (bottom) in lysates of ARPE19 cells transduced with the respective lentivirus. Abbreviations: kDa, kilo Daltons. (TIF) [file ppat.1012367.s002.tif]

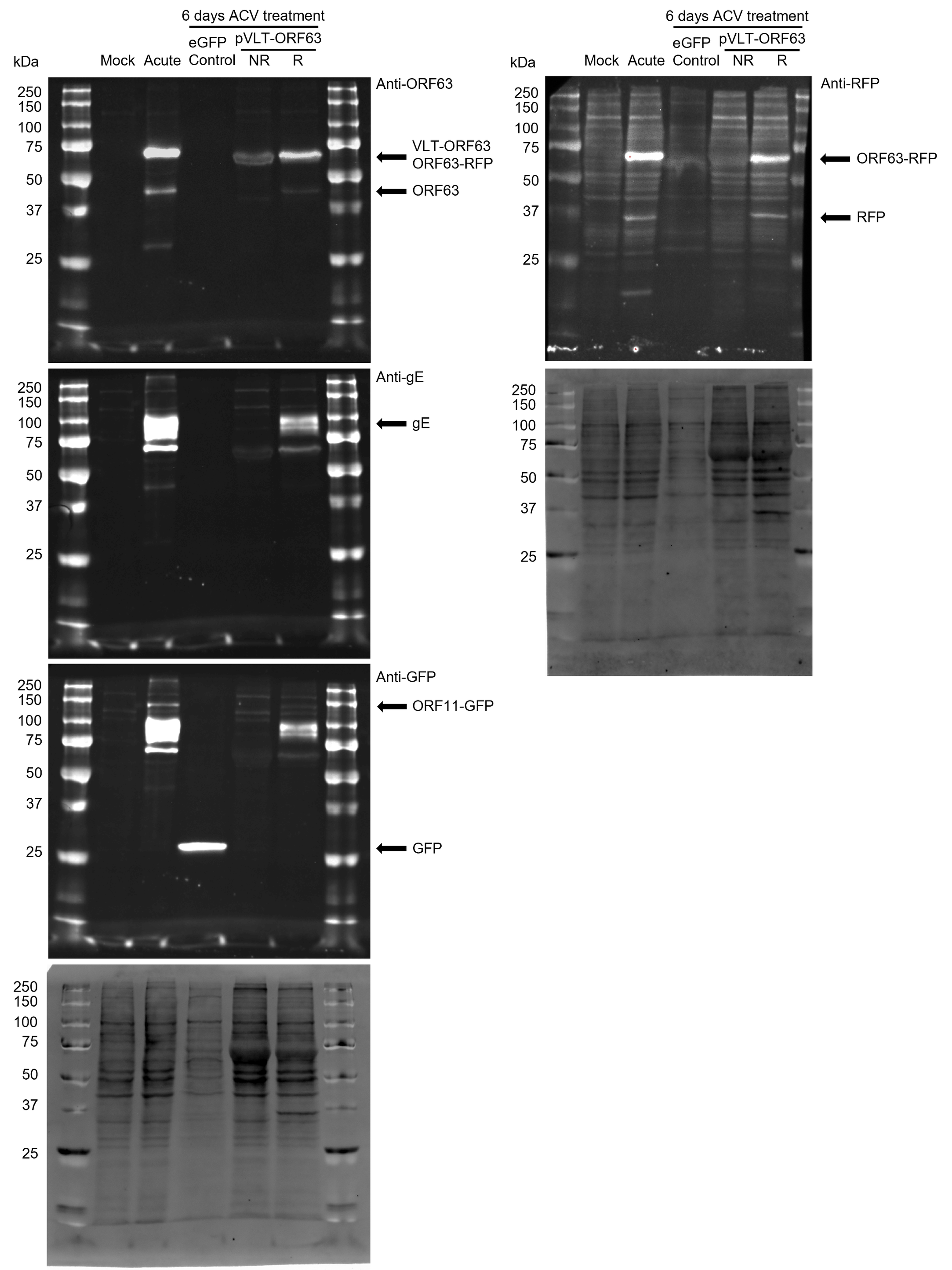

Supplement: S3 Fig — Western blots showing VZV proteins after incubation with the indicated antibodies and total protein detected with TCE staining (bottom blots) in lysates of dSH-SY5Y cells mock-infected or infected with v63R/11G in the absence (acute) or presence of ACV for 6 days and transduced with a lentivirus expressing eGFP or pVLT-ORF63. NR refers to “non-replicating” VZV, while R refers to “replicating” VZV, based on the lack or presence of ORF63-RFP/ORF11-GFP expression. Abbreviations: kDa, kilo Daltons; ACV, acyclovir. (TIF) [file ppat.1012367.s003.tif]

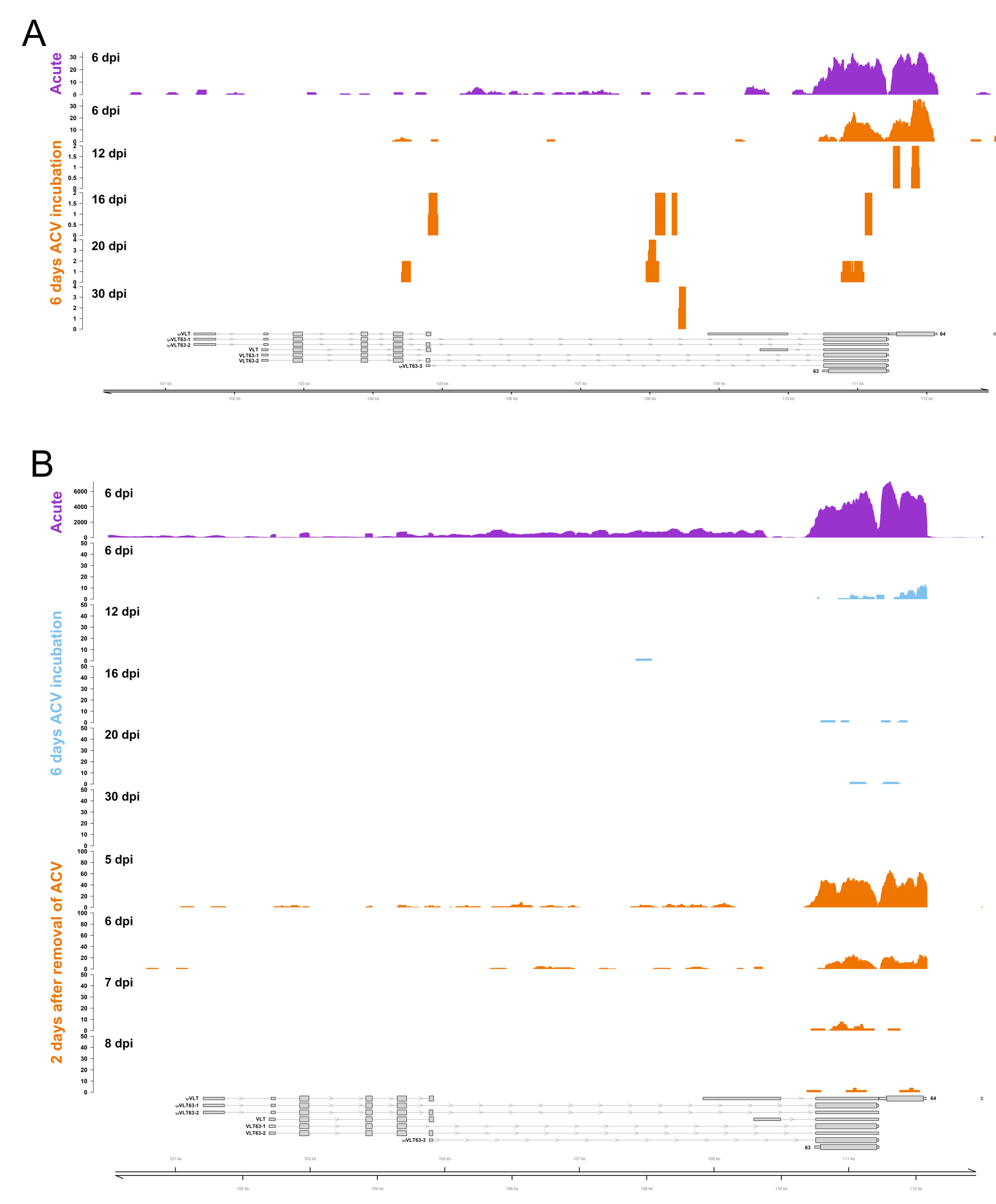

Supplement: S4 Fig — (A,B) Expanded views of transcription profiles in the VLT and ORF63 regions of dSH-SY5Y cells infected with v63R/11G in the absence (acute, violet) and presence of ACV for 6 dpi (orange, A; blue, B) or for 3, 4, 5 and 6 dpi in the presence of ACV with samples analyzed 2 days after ACV removal (orange, B). Bulk RNA-Seq was performed at different times post-infection, as labelled. Transcription from both DNA strands is shown with the depth of coverage labelled on the y-axis. A representation of the VLT and ORF63 regions is shown. Abbreviations: dpi, days post-infection; ACV, acyclovir. (TIF) [file ppat.1012367.s004.tif]

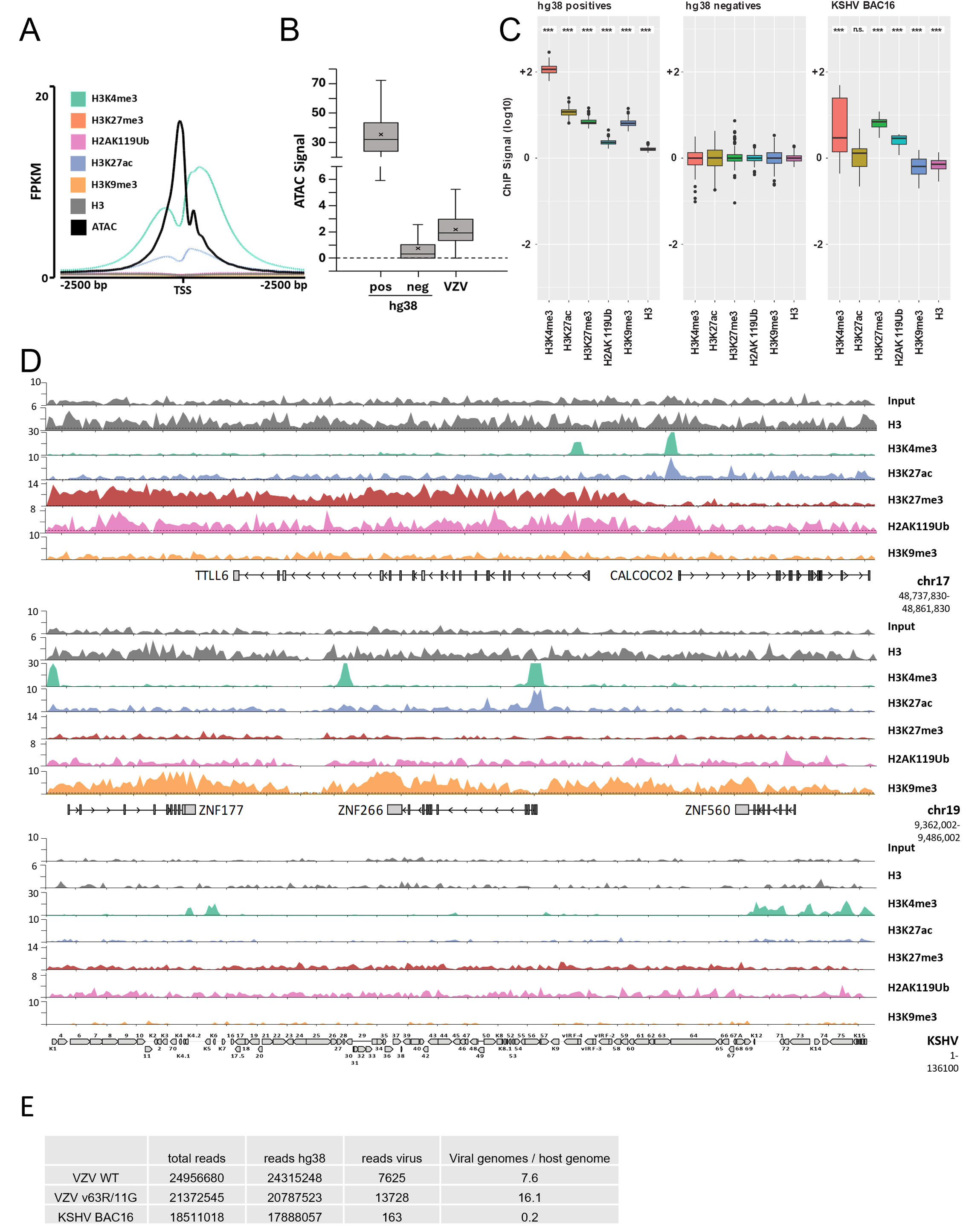

Supplement: S5 Fig — (A) Average read density of ChIP-seq and ATAC-seq reads at all human TSS (+/−2.5 kb) from dSH-SY5Y cells quiescently infected with VZV (VZV pOKA WT) at day 13 p.i. (B) Input-normalized quantification of ATAC-seq coverage at all positive host sites (n=54760) compared to a count/size-matched collection of randomly selected control regions and 500 bp sliding windows across the VZV genome (n=249) from dSH-SY5Y cells quiescently infected with VZV (VZV pOKA WT) at day 13 p.i. (C) ChIP seq data of dSH-SY5Y cells latently infected with KSHV (BAC16) at day 13 p.i. Input-normalized quantification of ChIP-seq signals in a 10 kb sliding window across the KSHV genome (KSHV BAC16, right panel), relative to the 200 most significantly enriched host regions (hg38 positives, left panel) and an equal number of size matched, randomly selected host control loci (hg38 negatives, center). Signals observed in host control regions were set to 1 (10E0). (P > 0.05 (n.s), P ≤ 0.05 (*), P ≤ 0.01 (**), P ≤ 0.001 (***) Wilcoxon-Mann-Whitney-Test of indicated sample compared to corresponding hg38 negatives). (D) Read density coverage tracks of histone marks on two host loci and KSHV, determined by ChIP-seq. (E) Number of read alignments of ChIP-input samples to human (hg38) and virus (VZV pOKA WT or KSHV BAC16). Number of reads were normalized to genome size (hg38 = 3.049 Gb, VZV = 124 kb, KSHV = 136 kb) and sequencing depth as RPKM values to estimate the fold enrichment of viral over host as an approximation of viral copy numbers per cell. Abbreviations: FPKM, fragments per kilobase per million mapped fragments; pos, positive; neg, negative; chr, chromosome; RPKM, reads per kilobase per million mapped reads. (TIF) [file ppat.1012367.s005.tif]
