# Supplementary material for: Repression of varicella zoster virus gene expression during quiescent infection in the absence of detectable histone deposition
Source: PLoS Pathog. 2025 Feb 10;21(2):e1012367. doi: 10.1371/journal.ppat.1012367 (PMC11838886; doi:10.1371/journal.ppat.1012367)
Supplement: S2 Table — The table summarizes the number of replicates, fields of view analyzed, VLT DNA positive cells, and total of DAPI-stained cells. The percentage of VLT DNA positive cells (4.9%) represents the proportion of VLT DNA positive cells within the total DAPI positive cells. (DOCX) [file ppat.1012367.s007.docx]

| Replicates | Field of view | VLT DNA^+^ | DAPI |
| --- | --- | --- | --- |
| 1 | 5 | 8 | 220 |
| 2 | 5 | 14 | 265 |
| 3 | 4 | 13 | 229 |
| Total | 14 | 35 | 714 |
| VLT DNA^+^ cells |  |  | **4.9%** |

**S2 Table:** Quantification of DNAscope results obtained at 20 dpi in dSH-SY5Y cells infected with VZV and incubated with ACV during 5 days. The table summarizes the number of replicates, fields of view analyzed, VLT DNA positive cells, and total of DAPI-stained cells. The percentage of VLT DNA positive cells (4.9%) represents the proportion of VLT DNA positive cells within the total DAPI positive cells.
