# Supplementary material for: Repression of varicella zoster virus gene expression during quiescent infection in the absence of detectable histone deposition
Source: PLoS Pathog. 2025 Feb 10;21(2):e1012367. doi: 10.1371/journal.ppat.1012367 (PMC11838886; doi:10.1371/journal.ppat.1012367)
Supplement: S5 Table — The table summarizes the number of replicates, fields of view analyzed, VLT RNA positive cells, and total of DAPI stained cells. The percentage of VLT RNA positive cells (1.0%) represents the proportion of VLT RNA positive cells within the total DAPI positive cells. (DOCX) [file ppat.1012367.s010.docx]

| Replicates | Field of views | VLT probe^+^ | DAPI |
| --- | --- | --- | --- |
| 1 | 7 | 6 | 491 |
| 2 | 5 | 4 | 382 |
| 3 | 6 | 4 | 473 |
| Total | 18 | 14 | 1346 |
| VLT^+^ cells |  |  | **1.0%** |

**S5 Table:** Quantification of RNAscope results obtained at 12 dpi in dSH-SY5Y cells infected with VZV and incubated with ACV during 6 days. The table summarizes the number of replicates, fields of view analyzed, VLT RNA positive cells, and total of DAPI stained cells. The percentage of VLT RNA positive cells (1.0%) represents the proportion of VLT RNA positive cells within the total DAPI positive cells
