# Supplementary material for: Repression of varicella zoster virus gene expression during quiescent infection in the absence of detectable histone deposition
Source: PLoS Pathog. 2025 Feb 10;21(2):e1012367. doi: 10.1371/journal.ppat.1012367 (PMC11838886; doi:10.1371/journal.ppat.1012367)
Supplement: Attachment — Submitted filename: 20250113__Rebuttal_Letter_SH-SY5Y_VZV.pdf [file ppat.1012367.s012.pdf]

Dear Editorial team,

Thank you very much for considering our manuscript for publication in PLoS Pathogens. We would like to thank the reviewers for taking the time to review our manuscript and for providing constructive criticism and comments. We have taken their suggestions seriously and addressed every one of their comments in a point-by-point response (see below). We believe that the result is an improved manuscript.

CC: rfkalejta@wisc.edu, dneumann3@wisc.edu, "Jiayi Wang" wang.jiayi@mh-hannover.de, "Nadine Brückner" brueckner.nadine@mh-hannover.de, "Simon Weißmann" simon.weissmann@leibniz-liv.de, "Thomas Günther" thomas.guenther@leibniz-liv.de, "Shuyong Zhu" shuyong_zhu@hotmail.com, "Carolin Vogt" vogt.carolin@mh-hannover.de, "Guorong Sun" sun.guorong@mh-hannover.de, "Renzo Bruno" renzonbruno@gmail.com, "Birgit Ritter" ritter.birgit@mh-hannover.de, "Lars Steinbrück" steinbrueck.lars@mh-hannover.de, "Benedikt Kaufer" benedikt.kaufer@fu-berlin.de, "Daniel P Depledge" depledge.daniel@mh-hannover.de, "Adam Grundhoff" adam.grundhoff@leibniz-liv.de

Dear Professor Viejo-Borbolla,

Thank you very much for submitting your manuscript "Repression of varicella zoster virus gene expression during quiescent infection in the absence of detectable histone deposition" for consideration at PLOS Pathogens. As with all papers reviewed by the journal, your manuscript was reviewed by members of the editorial board and by several independent reviewers. In light of the reviews (below this email), we would like to invite the resubmission of a significantly-revised version that takes into account the reviewers' comments.

All three reviewers found this work to be compelling, but agreed that additional characterization regarding the model was warranted. It is important to address each of the comments provided. In addition, the authors should carefully quantitate by multiple methods (qPCR and GFP expression) genomes in the quiescent cells. The reviewers all agree that there are alternative explanations for the lack of reactivation observed in the model, including no infection (or very low level of infection with VZV) and additional experimentation is warranted here to more conclusively support the conclusions presented. The authors should also provide statistical analyses for the data presented and consider performing microscopy experiments that show whether the quiescent genomes are trapped in PML bodies or capsids, two interesting hypotheses brought up in the future directions and discussion.

We cannot make any decision about publication until we have seen the revised manuscript and your response to the reviewers' comments. Your revised manuscript is also likely to be sent to reviewers for further evaluation.

When you are ready to resubmit, please upload the following:

[1] A letter containing a detailed list of your responses to the review comments and a description of the changes you have made in the manuscript. Please note while forming your response, if your article is accepted, you may have the opportunity to make the

peer review history publicly available. The record will include editor decision letters (with reviews) and your responses to reviewer comments. If eligible, we will contact you to opt in or out.
[2] Two versions of the revised manuscript: one with either highlights or tracked changes denoting where the text has been changed; the other a clean version (uploaded as the manuscript file).
Important additional instructions are given below your reviewer comments.
Please prepare and submit your revised manuscript within 60 days. If you anticipate any delay, please let us know the expected resubmission date by replying to this email.
Please note that revised manuscripts received after the 60-day due date may require evaluation and peer review similar to newly submitted manuscripts.
Thank you again for your submission. We hope that our editorial process has been constructive so far, and we welcome your feedback at any time. Please don't hesitate to contact us if you have any questions or comments.
Sincerely,

Donna M Neumann
Academic Editor
PLOS Pathogens

Robert Kalejta
Section Editor
PLOS Pathogens

Michael Malim
Editor-in-Chief
PLOS Pathogens
orcid.org/0000-0002-7699-2064

****

Reviewer's Responses to Questions

Part I - Summary
Please use this section to discuss strengths/weaknesses of study, novelty/significance, general execution and scholarship.

Reviewer #1: In this manuscript, Wang et al characterize a novel neuronal cell line based system for the study of VZV latency/quiescence. They discover the conditions necessary for differentiated SH-SY5Y to harbor VZV genomes in the absence of viral replication and near absence of viral transcription. Ultimately, these cells can be reactivated by direct addition of viral transcription activating proteins. Overall I think this is a well controlled paper documenting an interesting system and very interesting findings. I have only minor comments that I feel would improve the clarity and presentation of the findings.

Reviewer #2: In their study, Wang et al. explore the potential of the neuroblastoma-derived cell line SH-SY5Y as a model to investigate the mechanisms involved in the repression and subsequent reactivation of VZV gene expression. They demonstrate that differentiated SH-SY5Y (dSH-SY5Y) cells support productive infection by VZV. Treatment with acyclovir (ACV) inhibits viral replication and leads to a gradual repression of viral activity. After ACV removal, some cells produce viral particles, while others harbor non-replicating VZV genomes and transcripts containing VLT for at least 20 days post-infection (dpi). Introduction of exogenous VLT-ORF63 induces productive infection, indicating that the repressed genomes in non-replicating state remain capable of functionality. Histone deposition is notably absent at VZV genomes in quiescently infected dSH-SY5Y cells, suggesting a potentially novel mechanism of VZV repression in this neuronal context.
Developing neuronal models to study neurotropic herpesviruses is essential for understanding their biology, particularly latency and reactivation processes. In this context, the study by Wang et al. thoroughly explores the use of dSH-SY5Y cells as a potential additional cell culture model for investigating VZV latency. However, a significant limitation in this reviewer's opinion is the lack of a deeper investigation into the ACV model of VZV latency establishment. Specifically, the failure to achieve reactivation with LY294002 or SAHA does not align with previous studies by Markus et al. (PLoS Path, 2015). A more significant concern is the inability to detect histones and modified histone marks on the quiescent VZV in this model, despite these being described by Gary et al. (JVI, 2006) using human TG. The reviewer has confidence in the authors' proficiency in performing ChIP experiments. However, without using additional viral tools to establish a VZV latency-like state in dSH-SY5Y cells and conducting further ChIP, CUT&Run, or CUT&Tag analyses, it remains unclear whether the observed putative non-nucleosomal form of latent VZV is an intrinsic property of the virus or a bias introduced by using ACV. Consequently, there are major questions regarding the accurate use of this model. Without deeper mechanistic analyses, the study serves as a strong description of a cell culture model for investigating VZV latency but likely falls short in providing insightful answers to key questions about VZV biology.

Reviewer #3: Wang et al. present an interesting study investigating Varicella zoster virus (VZV) latency in human neurons. They develop a cost-effective model by modifying the neuronal differentiation protocol of the SH-SY5Y cell line to produce a predominantly neuronal-like cell type (dSH-SY5Y). Importantly, these cells can be infected with VZV and treatment with acyclovir (ACV) for 6 days post infection (dpi) can prevent induction of VZV genes while retaining low levels of VZV genomes within the cells. Interestingly, treatment with ACV for 5 dpi establishes a heterogeneous population of cells with around half exhibiting VZV replication and half exhibiting no VZV replication. This condition could be effective for studying how VZV latency is induced and controlled from both the host and virus side. Nevertheless, the authors focused on the ACV 6dpi treatment. The authors then use RNA-seq, ChIP-seq, and ATAC-seq to conclude that

there are quiescent VZV genomes that are free of histone deposition. While the premise is interesting, the study appears to be lacking statistical power that would be critical to evaluating their data. Similarly, oftentimes the authors overinterpret some of their results, specifically as relates to histones, and as such unfortunately disregard the underlying biology, raising questions about the impact of this study.

Part II – Major Issues: Key Experiments Required for Acceptance
Please use this section to detail the key new experiments or modifications of existing experiments that should be absolutely required to validate study conclusions.

Generally, there should be no more than 3 such required experiments or major modifications for a "Major Revision" recommendation. If more than 3 experiments are necessary to validate the study conclusions, then you are encouraged to recommend "Reject".

Reviewer #1: 1. For all images scale bars could be drawn thicker and larger. It would help to show the scale of the clusters of neural-like nuclei.

We have increased the size of the scale bars to shown the scale of the clusters.

2. In Figures 2 and 3 it is said the presence of both ORF63-RFP and ORF11-GFP denotes active replication. Especially in the ACV withdrawal experiments in Figure 3 are there ever any cells that are just ORF63-RFP positive which might be indicative of an early reactivation without progression to the late stage?

We observed some instances of ORF63-RFP without detectable expression of ORF11-GFP. However, in figure 3 we represented as positive for replication all cells that were RFP positive, indicative of at least start of replication, irrespective of whether they expressed or not detectable levels of GFP. We apologise for not being clear enough in the description of this figure. We have modified the text (lines 179-182 and 203-206).

3. Often the total number of experiments is displayed, but there is no mention of biological replicates that might be controlling for the batch effects with the 20-day neuronal differentiation culture experiments. In essence, in Figure 3 were the 6 dpi n=70 wells done with one batch differentiated neurons or accumulated over multiple independent experiments. Same with the 2 dpi n=8, etc. There are also multiple instance in the manuscript where the percentage of cells displaying a phenotype is mentioned in the text, but there is no accompanying figure that could also give key breakdowns of experiment to experiment variability. This should be rectified for Figure 4G (5% of cells genome positive, over how many biological replicates/fields of view), Figure 5H (4.4% of cells genome positive, at 12 dpi and 20 dpi?), and Figure 7C (% of cells with VLT RNA versus genome containing).

We apologise for the lack of such information in the original submission. We have modified the text and included extra panels and Supplementary Tables 1-6. The data shown in Figure 3 corresponds to four biological experiments consisting of four SH-SY5Y differentiations followed by infection in the presence of five different ACV incubation times (2-6 dpi ACV treatment). Each biological experiment contained two wells incubated with ACV during 2 dpi, as well as five wells incubated with ACV during 3, 4, 5 and 6 dpi. Then we focused on ACV incubation for 5 and 6 dpi and performed four and ten additional differentiation and infection experiments for each time point, respectively, each time with five wells. We have included in the figure legend how many biological repeats were performed and the number of analysed wells ("n") is shown in the graph.
We have included the requested information for figures 4G, 5H and 7C in Supplementary Tables 1-6. The images shown in Figure 4G correspond to 41 and 14 fields of view acquired randomly for 12 and 20 dpi, respectively, obtained from three biological experiments. In the case of Figure 5H, we took 41 and 16 random images for 12 and 20 dpi, respectively, from three biological experiments. Regarding Figure 7C, we cannot address how many cells that contain the genome express VLT RNA, because it is not possible to perform DNA and RNA scope in the same sample. Based on the percentages obtained when we detected the genome and transcript independently, we calculate that approx. 23% of the genome containing cells express VLT RNA.

4. For the ChIP-Seq experiments it is stated that all experiments were done with both WT and v63R/11G viruses, however there are only tracks shown for one replicate and Figure 8C is simply labeled Replicate 1 and 2. Please explain.
We apologise for the confusion. Numbers 1 and 2 do not refer to replicates, but to the two different viruses employed, pOka WT and v63R/11G viruses, corresponding to samples 1 and 2, respectively. We have modified the figure to include the names of the viruses employed.

5. The lack of any appreciable histone signal is a bold claim that appears to be supported by the internal matching cellular data. One additional piece of evidence supporting this claim might also already exist within the paired end ATAC-seq data: If the ATAC insert size between the two paired end reads is mapped you often get a periodicity of reads in multiples of ~160bp, i.e., the size of DNA wrapped around a nucleosome. Does the ATAC data for the human genome have this periodicity while the viral genome does not?

We thank the reviewer for this excellent suggestion. To allow a more detailed analysis, we increased the sequencing depth in the ATAC-Seq experiment by approximately 10-fold and compared fragment size distribution across the VZV and human genomes. The nucleosomal pattern of human fragment sizes was clearly visible, even when subsampled to the same number of fragments that aligned to VZV. In contrast, we could not detect such nucleosomal pattern for VZV, suggesting that the majority of viral genomes indeed does not contain nucleosomes. We explain these results in lines 391-399 and show them in Figure 8C (new experiment).

Reviewer #2: Here are some suggestions that could potentially enhance the content of the manuscript.
Major:
1) Reactivation experiments using LY294002 and SAHA were supposedly conducted at 34°C, though this is not specified in the Methods section. According to Markus et al., PLoS Path, 2015, which the authors reference, LY294002 appears more effective at 34°C for reactivating VZV in latently infected ESC-derived neurons. The authors should attempt these conditions before concluding the inefficacy of LY294002 and SAHA.

Thank you for the suggestion. We did not perform reactivation experiments at 34°C in the original submission. However, we agree with the reviewer that this could improve the chance of detecting reactivation. Therefore, we performed these experiments during the revision and did not detect reactivation with SAHA or LY294002 at this temperature. We mention this in lines 284-289 (new experiment).

2) Suppl Fig3: The "NR" samples express lower levels of pVLT-ORF63. Could their failure to reactivate be due to the ectopic pVLT-ORF63 levels being below a necessary threshold? How to address this issue? If a threshold aspect is to be considered, then the concluding sentence: "These results show that pVLT-ORF63 releases the repression on the VZV genome in dSH-SY5Y cells incubated with ACV for 6 days, highlighting that the infection was not abortive" should be revised to incorporate the potential influence of a pVLT-ORF63 threshold

We employed an anti-ORF63 antibody to detect pVLT-ORF63, since we do not have specific antibodies that would differentiate between ORF63 and pVLT-ORF63. Therefore, we do not know whether the signal detected in "R" and "NR" samples corresponds to pVLT-ORF63 or to ORF63-RFP, that have similar sizes (compare the bands in the blots detected with anti-ORF63 and anti-RFP). We think that the higher intensity of the band in "R" samples observed in the blot detected with the anti-ORF63 antibody corresponds to the combined protein expressed from the lentivirus pVLT-ORF63 plus ORF63-RFP expressed upon transduction and "de-repression". However, we cannot be certain about this. Therefore, we have included a sentence indicating that a potential influence of the pVLT-ORF63 threshold (lines 307-310).

3) It is difficult to believe that a DNA entity could remain in the nucleus in a non-nucleosomal form. However, this is what the data obtained by ChIP-seq and ATAC-seq appear to indicate. To my knowledge, the chromatinization state of VZV during infection is not well understood. However, Gary et al. (JVI, 2006) reported that the H3K9ac chromatin mark is associated with latent VZV genomes, at least on ORF62 and ORF63 in human TG, and on ORF62, ORF63, ORF36, and ORF14 during productive infection of MeWo cells. The investigation of this mark is missing in the Wang et al. study and should be included.

We agree that lack of detectable histone deposition is puzzling. We also do not understand how the VZV genome could remain in the nucleus without histone deposition. We also do

not claim that this the "normal" state of VZV during latency. It is also correct that Gary et al., detected H3K9ac in the VZV genomes obtained within 24 of death. We have included this in lines 489-491. H3K9ac modification is normally associated with active transcription. To have deposition of H3K9ac it is necessary that histone H3 is present. Since we do not detect histone H3, we consider that is not possible to have H3K9ac. Therefore, we have not performed this experiment.

Additionally, the results obtained by Wang et al. could be attributed to the latency model using ACV to artificially block viral replication and induce a latency-like state. However, numerous studies using ACV to establish an HSV latency-like state in cell culture models indicate that ACV does not prevent HSV chromatinization. However, the situation could be different for VZV. Figure 4 illustrates that early after infection in the presence of ACV, viral products such as ORF61 and ORF68 are expressed. These viral products or others present or not in the viral particle tegument could potentially impact viral chromatinization by influencing cellular chromatinization mechanisms in some way. If this is indeed the case, VZV would have evolved a very interesting putative mechanism to evade chromatinization and potentially avoid repression through this process. However, findings from Gary et al. suggest that such an assumption may not align with their observations. It is crucial to clarify this aspect by employing alternative methods to establish a latency-like state, such as using a replication-defective VZV mutated in an essential immediate-early gene, or UV-inactivated virus capable of infecting SH-SY5Y cells. Without such alternative approaches to study VZV latency, readers might perceive the absence of chromatinization of quiescent VZV under ACV as a limitation of the entire study. Significantly, the lack of reactivation observed with LY294002 and SAHA as depicted in Figure 6 could be attributed to the absence of a chromatinized state in this model. The low percentage of cells positive for VZV DNA, as depicted by FISH in Fig. 5H, may also be associated with the ACV-induced quiescent state of VZV and the reduction in viral genomes per cell, as measured in Figures 4F and 5G.

Our original hypothesis was that VZV contains histones during quiescent infection. We would like to emphasise that our objective was to study how VZV genomes are repressed prior to establishment of a latent infection. We achieved repression of VZV and persistence of viral genomes. However, we do not claim that we achieved latency as it is observed in human trigeminal ganglia. We still think that histone deposition and modifications are required for VZV latency, as shown for other herpesviruses including HSV and KSHV. Indeed, we show chromatinization of KSHV in our model (Supplementary Figure 5, new experiment), indicating that the results obtained were not dSH-SY5Y-dependent. KSHV chromatinization was observed despite having lower viral copy numbers relative to VZV (lines 426-430 and 497-501), indicating that our ChIP-seq is sensitive enough to reveal chromatinization even at low viral copy numbers, provided the genomes are chromatinized

The reviewer would like that we test other models such as infection with a VZV lacking expression of immediate early genes. We agree that this is an interesting approach. However, establishing such a model would basically imply repeating most of the experiments performed in this manuscript, delaying submission since they are very time

consuming. Therefore, we think that such experiments should be part of a different project and are beyond the scope of this revision. The hypothesis that certain VZV ORFs expressed prior to repression of gene expression could contribute to the observed phenotype is interesting. However, the gene expression observed is very low (approx. 2-logs less than actin) and we could not detect protein expression at those time points (not shown).

Reviewer #3: 1) The lack of viral genomes does not necessarily indicate no reactivation, it may just mean that there is no infection to begin with. It is unclear whether the 'clearing' of the viral genomes means they have latent infection or they are simply not infected if no viral genomes can be detected. Latent viral genomes are detectable in many systems (HPV, KSHV, EBV etc), which would be an important aspect of a functional latency model.

Infection of dSH-SY5Y takes place, because we can clearly monitor increased viral gene expression, replication and number of RFP- and GFP-positive cells with time in the absence of ACV, an infection that was performed in parallel, employing the same multiplicity of infection as in the infections carried out with ACV (Figures 2, 4, 5, 6, 7 and S1). Similarly, dSH-SY5Y infected in the presence of ACV for less than 6 dpi show VZV gene expression and spread in high percentage of wells upon ACV removal, showing that infection had occurred as well as spontaneous "reactivation" (Figure 3). The spread in several cellular clusters indicated initial infection of several cells, since VZV only spreads cell-to-cell in this and most in vitro cellular systems. We also detected viral genomes by qPCR and transcripts by RT-qPCR at different time points in samples treated with ACV for 6 days (Figure 5D-G). The genome copy number and viral transcripts decreased with time after infection in the presence of ACV for 6 days. We also detected the viral genomes by DNAscope at 12 and 20 dpi following incubation with ACV for 5 and 6 dpi in the presence of ACV (Figures 4G and 5H). Similarly, we detected many genomes in the acutely infected cells (shown in Figures 4G, 5H and in the image below (genomes detected by DNAscope are shown in pink, cell nuclei in blue):

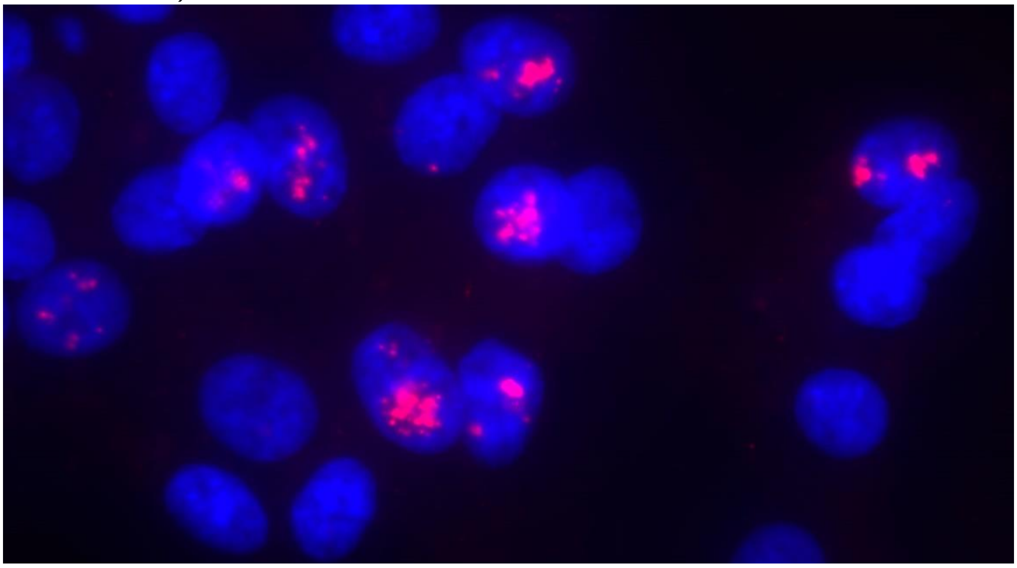

We also detected low level of genome-wide VZV transcription by RNA-seq in dSH-SY5Y cells infected in the presence of ACV for several days, including 6 dpi (Figure 7). Finally, we also obtained a sufficient input read coverage in the ChIP-seq and ATAC-seq samples. All these results show that there was infection of dSH-SY5Y and that ACV incubation for 6 dpi led to reduced genomes and transcripts and lack of spontaneous de-repression.

We are also aware that histones are detected on the latent genomes of other viral infections. As we indicated above, and as we carefully explained in the manuscript, we do not claim that we have latent genomes as detected in human sensory ganglia lacking histones, but we claim that there is a mechanism that represses VZV in the absence of chromatin deposition and that some of these genomes are viable. To address whether a similar phenomenon would be observed for other herpesviruses, we infected dSH-SY5Y with KSHV BAC16 in the presence of ACV during 13 dpi and compared the histone deposition on VZV with that on KSHV (lines 413-430, Supplementary Figure 5, new experiment). We chose KSHV because we have previously characterized by ChIP-Seq the whole genome histone deposition on this virus (Gunther and Grundhoff, 2010, PLoS Pathogens, DOI: 10.1371/journal.ppat.1000935). Interestingly, we detected histone deposition on the KSHV genomes, while we did not detect this on the VZV ones. The profile of histone deposition on KSHV was similar to that detected previously upon latent infection of endothelial cells (Gunther and Grundhoff, 2010, PLoS Pathogens, DOI: 10.1371/journal.ppat.1000935). We compared the copy numbers based on read coverage from ChIP-input samples. Based on these results, there were fewer viral copies of KSHV BAC16 genomes per human genome than for VZV. These results suggest that the lack of histone deposition observed was not due to a low copy number of VZV genomes (discussed in lines 497-501). Our results suggest that most of VZV DNA is not associated with chromatin in this model.

Also, it is difficult to compare the findings between groups. For example in figure 3, genomes present in these wells measured by qPCR would be more compelling than simply not RFP/GFP positive, and from there a ratio of which wells have detectable VZV genomes to RFP/GFP would enable validation of virus in the wells and a more convincing latency model. Also figure 4B and 5B would need the VZV genome staining to confirm the cells displayed are latently infected and not just uninfected.

We thank the reviewer for this suggestion. However, the data shown in figure 3 corresponds to 4, 8 and 14 biological experiments (differentiation plus infection) for samples treated with ACV for 2-4 dpi, 5 dpi and 6 dpi, respectively. Differentiation takes 18 days and each infection would take up to 30 days of observation, as we depict in the graphs in Figure 3. To repeat such an experiment to perform RT-qPCR plus microscopy analysis of the cells would dramatically delay this submission without adding, in our humble opinion, more information to that shown in Figure 4F and 5G, where we depict the genome copy number of "non-replicating" and "replicating" wells (corresponding to RFP/GFP negative and positive, respectively). Therefore, we hope that the reviewer will understand if we consider such experiments not strictly required for the current manuscript.

Regarding the second point, our results in Figures 4B and 5B show representative images of at least 40 differentiations in which we followed up the reactivation or not of VZV. In several of these differentiations, we detected the genome by in situ hybridisation. This was already shown in figures 4G and 5H, where we showed VZV genomes in non-reactivated wells. We also quantified the VZV genomes by qPCR (Figures 4F and 5G). These results clearly show that non-reactivated (NR) wells contained dSH-SY5Y cells that were infected.

2) The conclusions based on the number of viral genomes detected are highly problematic. There are several reasons for this concern: 1) they detect viral genomes in only 5% of the cells, which is more indicative of no infection that latency, as stated above;

We agree that we have a low level of infection. This is due to the low multiplicity of infection employed because it is cumbersome to obtain high titres of cell-free VZV. We employed an MOI of 0.001. Therefore, having 5% of cells positive for the viral genome by DNAscope at days 12 and 20 pi, following infection in the presence of ACV during 6 dpi is not indicative of no infection. It is indicative of the level of infection that we can achieve when VZV DNA replication is blocked with ACV. Moreover, expression of pVLT-63 induces replication and VZV spread, indicating that some dSH-SY5Y cells were infected with viable genomes

2) The conclusion that there are no histones on the viral genomes based on their findings is not convincing. The lack of statistical analysis also suggests that these datasets were not thoroughly analyzed, especially when the data presented indicate that there are peaks of histones (eg H3K27me3 and H3K9me3) that with different analyses and greater signal would be apparent.
Given the biology that all known latent viruses have repressive chromatin deposited, it is a higher burden of proof to say the opposite, which would require more systematic approaches and analysis than just lack of detection. For example, if the authors had a higher percentage of detectable latent genomes, then statistical power would support their conclusions. However, with such low numbers, lack of signal is not the same as saying histones are not present.

We appreciate the reviewer's concern regarding the analysis of histone modification enrichment. We are very much aware of the fact that analysis of viral chromatin poses unique challenges and would like to point out that our analysis was performed with a validated pipeline that was explicitly designed to evaluate histone modification enrichment on viral genomes (see Gunther et al., 2019, PLoS Pathogens, doi: 10.1371/journal.ppat.1007838). In addition to employing standard peak calling algorithms, the pipeline quantifies input-normalized ChIP-seq read counts and allows direct statistical evaluation of viral signals relative to the most significantly enriched host peaks and negative host control regions. By doing so, we can detect viral regions that significantly deviate from background; even in cases (such as this) where conventional peak-finding algorithms such as MACS2 and EPIC2 fail to detect significant peaks on the viral genome.

We thus opine that our analysis is substantially more thorough than approaches employing standard peak callers only.

Our results clearly show that, across the entire viral genome, VZV ChIP-seq reads are significantly underrepresented, even when compared to negative host control regions. As this is also true for a pan-H3 antibody, we think it most logical to conclude that most viral genomes lack canonical chromatin. Importantly, this result is not due to viral genome counts generally being below the limit of detection, as quantification of input reads show an average copy number that is well above that of host loci (see Suppl. Figure S5E). The absence of fragment-length periodicity in viral ATAC-seq reads (revised Fig. 8C) independently supports the conclusion that most viral genomes lack nucleosomes. In a proof-of-principle experiment, we now also show that infection of dSH-SY5Y cells with KSHV yields the expected histone modification patterns (i.e., a largely polycomb-repressed genome with latently transcribed loci showing activating histone marks), even though viral copy numbers are 40-80 times lower than those observed with VZV (see Supplementary Figure 5, new experiment).

We apologize if our bioinformatics approach was not made sufficiently clear in the original manuscript and have added a paragraph to describe the methodology in greater detail (starting in line 778). Briefly, host regions were analysed using standard peak-finding methods to establish positive and negative control regions. For viral genomes, we subsequently utilized a segmentation approach, assessing enrichment via normalized ChIP-to-input read count ratios in sliding windows across the viral genome. These ratios were statistically compared to the negative controls using a Wilcoxon-Mann-Whitney test. We apologize for the fact that the corresponding box plots in the original Figure 8 did not show levels of statistical significance and have modified the revised Figure 8B and new Suppl. Figure 5C accordingly.

As already mentioned, we do not claim to have latency as would occur in sensory neurons in vivo. While the bulk of viral genomes persisted for more than 20 dpi without detectable deposition of histones, we also show that at least some of the genomes were viable because exogenous expression of VLT-63 led to production of infectious VZV. As discussed in the manuscript, however, we presently cannot exclude the possibility that the reactivated fraction stems from a minor population of chromatinized episomes that is masked by the bulk population (lines 532-535).

One possible experiment could be to stain for genomes by FISH in a cell that expresses an H3-GFP or similar tagged histones to look for co-localization, as has been done in other systems;

We thank the reviewer for this suggestion. We agree that co-localization studies can provide additional evidence especially in cases where histone deposition is evaluated by ChIP-qPCR, as has been done for the H3.3 variant during HSV-1 infection. However, such experiments can only provide circumstantial evidence, as they lack the resolution to distinguish between nucleosomal and free histones. In contrast, ChIP-seq (and related)

methods are the gold standard for direct and genome-wide interrogation of protein-DNA interactions, and our analysis of the host epigenome clearly demonstrate the validity of our ChIP-seq experiments and associated bioinformatic analyses. We therefore think that, even if there were significant microscopic co-localization, our conclusion that the majority of viral genomes are nucleosome-free would not change. Additionally, while variant histone H3.3 is only present at a subset of host regions and thus may be amenable to co-localization studies, the global host chromatin background makes similar studies with canonical H3.1 extremely challenging. We therefore hope that the reviewer will understand if we consider such experiments beyond the scope of the current manuscript. However, we plan to perform a follow-up study to evaluate co-localization of VZV genomes with different sub-nuclear structures and may also include histones in these experiments.

3) If the conclusion that histones are not present were true, then the authors should suggest a different model for repression or lack of gene expression. It has been well established that open chromatin or nucleosome-free regions are highly expressed, not silent;

Transcriptionally active chromatin is generally open, but to our knowledge the reverse is not necessarily true. While open chromatin is considered transcriptionally permissive, high-level transcription additionally requires that activator proteins such as transcription factors are present and able to bind to their cognate sequences. The term 'open chromatin' commonly suggests that the underlying DNA is readily accessible to transcription factors, but we do not argue this being the case here. We indeed find it very unlikely that, without being compartimentalized or otherwise shielded, a DNA molecule of more than 100 kb could persist in a non-chromatinized state for any extended period of time. We therefore hypothesize that membraneless compartments such as PML-NB or factors like Smc5/6 could be involved in the sequestration and silencing of VZV genomes. As mentioned in our answer to point 2 above, some of these genomes may reactivate upon VLT-63 expression, but we also cannot exclude the possibility that the reactivating fraction originates from a small population of canonically chromatinized genomes. While we think that these possibilities are adequately discussed in the current manuscript (lines 510-527), solving these questions will ultimately require a significant number of additional experiments in this and other systems that will be the subject of a follow-up study.

4) Another concern in the analysis is the comparison to input and the host genome. Because the viral genome is much smaller in comparison to the host, and the input sample is much higher in concentration by definition, then it is expected that the reads would be low. A positive control for a protein known to be bound to the viral genomes would be critical here to ensure that the ChIP is an accurate measure of proteins bound to the viral genomes. The text on page 16 and figure 8c describes positive and negative controls on the host genome, but no positive control for the viral genome was used, which calls into question whether the ChIP of viral genomes was successful enough to be interpreted. Similarly, if the genome lacked any periodicity of bound proteins, the ATAC-seq signal should be uniformly high, however, there are peaks and patterns in the ATAC-

seq data. Further experimentation and development of the model on viral genomes is required to draw conclusions.

We appreciate the reviewer's concern regarding genome size differences and the use of positive controls for the viral genome. Our analysis accounts for genome size, sequencing depth, and viral copy number by normalizing read counts to window size and input reads. This ensures that differences in genome scale and input representation are appropriately addressed, enabling direct comparison between host and viral regions. The details of this methodology are now included in the Methods section.
To further validate our approach, we applied the same quantification strategy to KSHV, and detected robust chromatin patterns despite its lower viral copy numbers (Supplementary Figure 5C-E). These results confirm that our method is sensitive enough to detect chromatinization on viral genomes and supports the validity of our ChIP-seq analysis.
Regarding the peaks and patterns in the ATAC-seq data, such features can also arise from stochastic uneven coverage or sequence bias rather than true periodicity of bound proteins. The weak peaks in some of the histone modification ChIP-seq experiments also very likely represent such background signals. Furthermore, our demonstration that there is no periodicity in the fragment size of ATAC-seq signal on viral-mapped reads (see Figure 8C and reply to reviewer #1, major point 5) aligns with the hypothesis that the majority of viral genomes are not chromatinized.
To our knowledge, there is no protein that is known to bind to VZV genomes during latency and thus could serve as a viral positive control. However, in our opinion, there is no reason to suspect that ChIP-seq would fail specifically on VZV genomes, especially with validated ChIP-seq antibodies that perform very well (as evidenced by the results obtained for the host and KSHV genomes). In view of this, absence of canonical chromatinization of the majority of viral genomes is the most likely explanation for the observed results.

3) Quantification of in situ hybridization data is included in the text but no quantification is provided in the figures. Also controls such as Mock infected and 1 or 6 dpi infection are missing.

We apologise for the lack of this information. We have added a graph in figures 4G, 5H and 7C representing the number of cells analysed and those that were positive for VZV DNA or VLT/VLT-63. We have also included tables compiling all the results from the different in situ hybridization experiments (Supplementary Tables 1-6). We show Mock-infected cells and cells at 6 dpi in the absence of ACV. We also performed DNAscope at different times post-infection. At day 1 post-infection many genomes (in red) are located in the cytoplasm (see below):

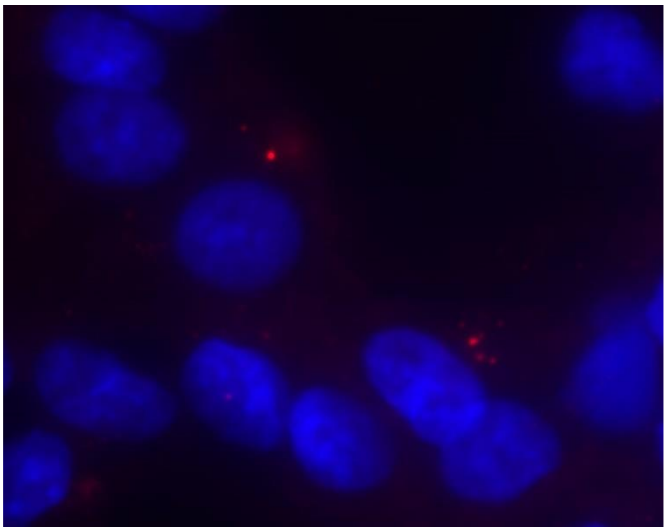

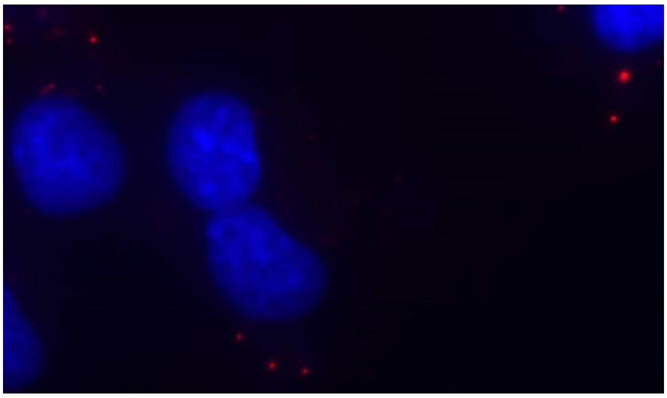

Part III – Minor Issues: Editorial and Data Presentation Modifications
Please use this section for editorial suggestions as well as relatively minor modifications of existing data that would enhance clarity.

Reviewer #1: 1. I find Days Post Infection and Days Post release confusing when discussing the NR wells versus the R wells, as both conditions could be said to be either DPI or DPR regardless of whether they start replicating or not. Maybe this could be made more clear in Figure 4A?
We apologise for the confusion. We have included the information on the days post-infection and days post-release in figure 4B. We hope this is clearer now.

Reviewer #2: 1. Fig3B: The abscissa indications of graph B are confusing and do not align with the explanations provided in the text.
We have changed the abscissa text in panels B and C to "Days in the presence of ACV after infection". We hope this is clearer now.

2. Figure 4B: The annotation "R" with the indication "5 days ACV treatment" is confusing, as it is similar to the "R" present in panels D, E, F, which pertains to days post-release from ACV treatment.
We apologise for the confusion. We have included the information on the days post-infection and days post-release in figure 4B. We hope this is clearer now.

3. Figures 4F and 5G show a decrease in the amount of VZV genomes with time up to 30 dpi. How do the authors explain this?
This is an interesting question that we cannot answer. We think that at day 2 post-infection (Figure 4F) we detect non-nuclear genomes (see answer to "Major Issue" number 3 from reviewer 3 above, DNAscope images showing cytoplasmic genomes at 1 dpi), that have not been targeted by ACV and potentially also genomes from the inoculum. At later time points, ACV inhibits replication of nuclear genomes and there is no detection of genomes from the inoculum. We do not know what is the fate of these genomes, maybe they are degraded due to lack of chromatinization in our model. However, interestingly, a few genomes persist in these conditions.

4. Figure 4A, 5A, 6A: The timeline lacks the indication of the days post-infection (dpi) when cells are analyzed.
We have included this information

5. The message conveyed by Figures 5 adds incrementally to Figure 4. Figures could be merged, or Figure 5 could be included in the supplementary figures.
We appreciate the comment but we think this will add confusion to the reader since in figure 4 we observe both repression and de-repression after removal of ACV (5 days ACV treatment post-infection), while in figure 5 we observed complete repression of VZV after 6 days ACV treatment post-infection. We also think that both figures should be maintained as main figures but we are willing to include one of them as supplementary.

6. Figures 4F, 5G: How do the authors account for the decrease in VZV genomes per cell? Is this linked to the ACV model of latency establishment? Could it be related to the absence of chromatinized quiescent genomes under this treatment?
This is an interesting question that we cannot answer. See answer to "Minor Issue" #3 above. It could be related to the lack of chromatinization in this model that leads to degradation of most genomes. This is also why it is interesting to find out that some genomes remain in the nucleus after 20 dpi in these conditions. After 6 dpi the number is relatively constant (going from 50 to 100 genomes / 100 cells, based on qPCR data) and some genomes persist for many days.

7. Figure 5C: IE4 and other IE, E proteins staining should be examined at earlier times post-infection (pi), starting from 2 dpi, to investigate whether IE proteins are produced during the period between 2 and 6 dpi.
We could not detect IE4 and other viral proteins at earlier time points infection.

8. Legend Figure 7: The figure description does not fully correspond with the visual content presented.
9. Figure A: what are the samples 12, 16, 20, 30 dpi?
These samples correspond to transcriptomic analyses of dSH-SY5Y cells infected with VZV in the presence of ACV during the first 6 days of infection. Total RNA was extracted on days 12, 16, 20 and 30 post-infection and subjected to RNA-sequencing

10. Figure B: The legend should be revised to accurately explain the representation of 5, 6, 7, and 8 dpi in orange, as it currently refers to 3, 4, 5, and 6 dpi. The legend should clarify the timescale of ACV addition/removal.
We apologise for the confusion. The samples represented as 5, 6, 7 and 8 dpi correspond to cells that were infected in the presence of ACV during 3, 4, 5 and 6 days and were processed for RNA extraction two days after removal of ACV (i.e., 5, 6, 7 and 8 dpi). We have modified the legend to clarify this.

Typos
We thank the reviewer for pointing all these typos.
P3l65: change ''infected cell polypeptide 0'' by '' 'infected cell protein 0'
We have performed this change
P55l1145-47, Figure 2 legend: change (B) by (C); (C-E) by (D-F); and (B-E) by (C-F)
We apologise for this mistake. We have corrected this.
P9l186-87: ''These results indicated that the time of ACV incubation positively correlates with the duration of VZV inhibition''. This correlation is not clearly supported by the findings in Figure 3. Further clarification or rephrasing may be necessary in the text to better explain this relationship
We have removed this sentence from the manuscript.

P10l194: Fig4B is not referred in the text.
We apologise for this mistake. We have included a reference to figure 4B in the text (line 216).

P15l315: change:'' the top and center panels in Figure 8'' by '' the top and center panels in Figure 8A''
We apologise for this mistake. We have performed this change

P15l319: change H3K3me3 by H3K4me3
We have performed this change

Reviewer #3: 1) Figure 1 and associated text: it is unclear how many differentiation tests this is from. Statistical analysis is needed.
We have performed more than 100 differentiations of SH-SY5Y cells, for the experiments shown in this manuscript and related projects. The success rate is higher than 95%. The results shown in Figure 1 correspond to 3 independent differentiations.
2) Multiple breaks in a graph are difficult to compare across samples. A log scale would be easier to follow.
We have modified the graphs and we show now log scales
3) Citations of figure 4 b-d are incorrect in the text between lines 216-221.
We thank the reviewer for this comment. We have corrected this
4) Figure 3 has a huge range of N for each time point (from 2 to 70), but this is not provided in the other figures. Again, thorough statistical analysis is needed to draw conclusions.

We apologise for the lack of this information. The data shown in Figure 3 corresponds to four biological experiments consisting of four SH-SY5Y differentiations followed by infection in the presence of five different ACV incubation times (2-6 dpi ACV treatment). Each biological experiment contained two wells incubated with ACV during 2 dpi, as well as five wells incubated with ACV during 3, 4, 5 and 6 dpi. Then we focused on ACV incubation for 5 and 6 dpi and performed four and ten additional experiments for each time point, respectively, each time with five wells. We have included in this figure legend and others the "n".

PLOS authors have the option to publish the peer review history of their article (what does this mean?). If published, this will include your full peer review and any attached files.

If you choose "no", your identity will remain anonymous but your review may still be made public.

Do you want your identity to be public for this peer review? For information about this choice, including consent withdrawal, please see our Privacy Policy.
Reviewer #1: No
Reviewer #2: No
Reviewer #3: No

Figure Files:
While revising your submission, please upload your figure files to the Preflight Analysis and Conversion Engine (PACE) digital diagnostic tool, https://pacev2.apexcovantage.com. PACE helps ensure that figures meet PLOS requirements. To use PACE, you must first register as a user. Then, login and navigate to the UPLOAD tab, where you will find detailed instructions on how to use the tool. If you encounter any issues or have any questions when using PACE, please email us at figures@plos.org.
Data Requirements:
Please note that, as a condition of publication, PLOS' data policy requires that you make available all data used to draw the conclusions outlined in your manuscript. Data must be deposited in an appropriate repository, included within the body of the manuscript, or uploaded as supporting information. This includes all numerical values that were used to generate graphs, histograms etc.. For an example see here on PLOS Biology: http://www.plosbiology.org/article/info%3Adoi%2F10.1371%2Fjournal.pbio.1001908#s5.
Reproducibility:
To enhance the reproducibility of your results, we recommend that you deposit your laboratory protocols in protocols.io, where a protocol can be assigned its own identifier (DOI) such that it can be cited independently in the future. Additionally, PLOS ONE offers

an option to publish peer-reviewed clinical study protocols. Read more information on sharing protocols at https://plos.org/protocols?utm_medium=editorial-email&utm_source=authorletters&utm_campaign=protocols

---

In compliance with data protection regulations, you may request that we remove your personal registration details at any time. (Remove my information/details). Please contact the publication office if you have any questions.
